# Sodium Bicarbonate Treatment during Transient or Sustained Lactic Acidemia in Normoxic and Normotensive Rats

**DOI:** 10.1371/journal.pone.0046035

**Published:** 2012-09-28

**Authors:** Franco Valenza, Marta Pizzocri, Valentina Salice, Giorgio Chevallard, Tommaso Fossali, Silvia Coppola, Sara Froio, Federico Polli, Stefano Gatti, Francesco Fortunato, Giacomo P. Comi, Luciano Gattinoni

**Affiliations:** 1 Dipartimento di Anestesia, Rianimazione (Intensiva e Subintensiva) e Terapia del Dolore, Fondazione IRCCS Ca' Granda, Ospedale Maggiore Policlinico, Milan, Italy; 2 Centro di Ricerche Chirurgiche Precliniche, Fondazione IRCCS Ca' Granda, Ospedale Maggiore Policlinico, Milan, Italy; 3 Centro Dino Ferrari, Dipartimento di Scienze Neurologiche, Fondazione IRCCS Ca' Granda, Ospedale Maggiore Policlinico, Milan, Italy; 4 Università degli Studi di Milano, Milan, Italy; Harvard Medical School, United States of America

## Abstract

**Introduction:**

Lactic acidosis is a frequent cause of poor outcome in the intensive care settings. We set up an experimental model of lactic acid infusion in normoxic and normotensive rats to investigate the systemic effects of lactic acidemia per se without the confounding factor of an underlying organic cause of acidosis.

**Methodology:**

Sprague Dawley rats underwent a primed endovenous infusion of L(+) lactic acid during general anesthesia. Normoxic and normotensive animals were then randomized to the following study groups (n = 8 per group): S) sustained infusion of lactic acid, S+B) sustained infusion+sodium bicarbonate, T) transient infusion, T+B transient infusion+sodium bicarbonate. Hemodynamic, respiratory and acid-base parameters were measured over time. Lactate pharmacokinetics and muscle phosphofructokinase enzyme's activity were also measured.

**Principal Findings:**

Following lactic acid infusion blood lactate rose (P<0.05), pH (P<0.05) and strong ion difference (P<0.05) drop. Some rats developed hemodynamic instability during the primed infusion of lactic acid. In the normoxic and normotensive animals bicarbonate treatment normalized pH during sustained infusion of lactic acid (from 7.22±0.02 to 7.36±0.04, P<0.05) while overshoot to alkalemic values when the infusion was transient (from 7.24±0.01 to 7.53±0.03, P<0.05). When acid load was interrupted bicarbonate infusion affected lactate wash-out kinetics (P<0.05) so that blood lactate was higher (2.9±1 mmol/l vs. 1.0±0.2, P<0.05, group T vs. T+B respectively). The activity of phosphofructokinase enzyme was correlated with blood pH (R2 = 0.475, P<0.05).

**Conclusions:**

pH decreased with acid infusion and rose with bicarbonate administration but the effects of bicarbonate infusion on pH differed under a persistent or transient acid load. Alkalization affected the rate of lactate disposal during the transient acid load.

## Introduction

Lactic acidosis is a frequent cause of acidemia in the intensive care settings, often associated with hemodynamic and/or respiratory impairment [1]–[9].

Whether it is worth to correct acidemia by infusion of alkaline solutions is a matter of discussion [10]–[12]. The evidence in favour of pH correction of organic acidemia is poor. Clinical studies are few and inconclusive, particularly with respect to clinical outcome [13]; [14]. There are a number of evidences against alkalinization therapy [14]–[23]. International guidelines “recommend against the use of sodium bicarbonate therapy for the purpose of improving hemodynamics or reducing vasopressor requirements in [septic] patients with hypoperfusion-induced lactic acidemia with pH>7.15” [24]. However, the correction of acidemia is common in clinical practice. An on line survey has recently shown that 67% of critical care physicians start to administer alkaline solutions to patients with lactic acidosis when pH is 7.2 [25].

Many experimental models of lactic acidemia do not allow to investigate the effects of a lactic acid load per se, because of confounding factors. Moving from the consideration that in clinical setting lactic acidosis may be transient, as during reperfusion of ischemic regions, or sustained, as in persistent hemodynamic instability, we decided to investigate the effects of bicarbonate infusion in normoxic and normotensive animals subjected to transient or sustained lactic acid load.

We wish to report here the results of our investigation and discuss the possible underlying mechanisms and clinical implications.

## Materials and Methods

This experimental study was performed after the Ethics Committee of our institution and the Italian Ministry of Health approved the protocol (Permit Number: 6/07). All surgery was performed under anesthesia, and all efforts were made to minimize suffering.

### Experimental design

**Figure 1:**
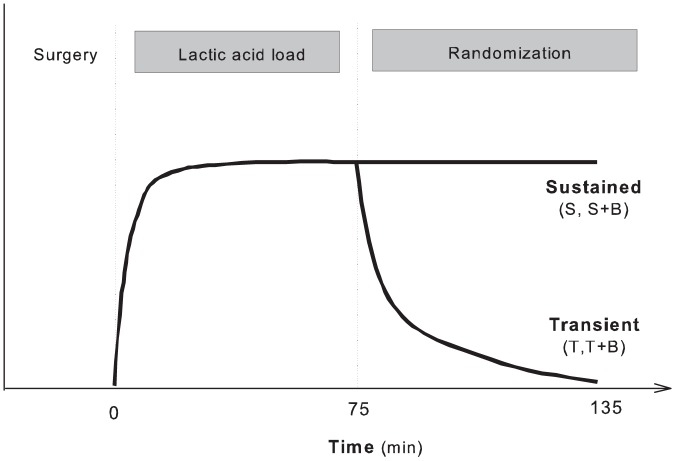
Protocol overview. A schematic overview of the experiment flow is shown in the figure. The investigation consisted of an initial lactic acid load to induce lactic acidemia followed by randomization to sustained (S) or transient (T) lactic acid infusion with or without sodium bicarbonate (B) treatment.

A schematic overview of the experiment flow is shown in Figure 1.

The investigation consisted of an initial lactic acid load to induce lactic acidemia followed by randomization to sustained or transient lactic acid infusion with or without sodium bicarbonate treatment.

### Anaesthesia and animal preparation

Sprague Dawley rats (weight 250–300 grams) purchased from Charles River, housed in a warmed and humidified ambient with a 12/12 hours day/night shift, received an intraperitoneal injection of 80 mg/kg thiopental. The trachea was cannulated with a 14 gauge tube connected to a pressure transducer (Motorola MPX 2010DP, Phoenix, AZ, USA). Paralysis was obtained with vecuronium bromide 3 mg/kg i.v. Right carotid artery, femoral and subclavian vein were cannulated with 22 gauge catheters. The arterial catheter was connected to a pressure transducer (Bentley Trantec 800, Santa Ana, CA, USA). Blood pressure and airway pressure were continuously monitored and digitally stored (Elekton Colligo, Agliano, AT, Italy) for subsequent analysis.

During the surgical preparation, rats were mechanically ventilated (Harvard-Rodent 683, Harvard Apparatus South Natick, Massachusetts, USA), with a tidal volume of 6 ml/kg, PEEP of 3 cmH_2_O and respiratory rate set according to mixed expired CO_2_ (mixCO_2_), continuously analyzed (Ohmeda 5250 RGM, Ohmeda, Louisville, CO, USA). At the end of the procedure blood was drawn from the arterial line for blood gas analysis (1620 pH/Blood Gas Analyzer and 682 CO-Oxymeter, Instrumentation Laboratory, Lexington, MA, USA). Respiratory rate was set to obtain the desired values of PaCO_2_ and pH. Inclusion criteria were: pH 7.35–7.45, PaCO_2_ 35–45 mmHg, lactate <2 mmol/l, hemoglobin >12 g/dl, rectal temperature >36°C, mean arterial pressure (MAP) >90 mmHg. Ventilator parameters remained unchanged throughout the protocol.

If no major problems occurred during surgical preparation and after stabilization time animals were included into the study.

### Lactic acid load and randomization process

After confirming inclusion criteria, 14.45 mmol/kg of a 0.55 M solution of L(+) lactic acid (30% in H_2_O by weight - CH_3_CH(OH)CO_2_H- Sigma Aldrich) was infused over 75 minutes through a catheter positioned in a central vein. Animals that met inclusion criteria (pH<7.3, lactate >3 mmol/l and mean arterial pressure >70 mmHg) after the acid load, were randomized by sealed envelopes to one of the following treatments (n = 8 animals per group): S) sustained infusion of lactic acid, S+B) sustained infusion+sodium bicarbonate, T) transient infusion of lactic acid, T+B) transient infusion+sodium bicarbonate. In the sustained groups (group S and S+B), lactic acid was infused throughout the protocol at a rate of 0.20 mmol/kg/min. In the transient groups (groups T and T+B), an equal amount of normal saline was infused. In animals randomized to bicarbonate infusion (groups S+B and T+B) a 1 M solution of sodium bicarbonate was infused at a rate of 0.137 mmol/kg/min; bicarbonate infusion rate was chosen according to pilot studies that suggested a lactate to bicarbonate infusion ratio of 1 M ∶ 0.7 M. If bicarbonate was not infused (groups S and T) an equal volume of normal saline was given to the animals. Experiments were interrupted 60 minutes after randomization (135 minutes after the beginning of the infusion of acid lactic) or if animals developed severe and fatal hypotension.

### Outcome measurements

#### Acid-base parameters

Acid base parameters included measured (pH, pCO_2_), and calculated (HCO_3_, BE) variables (1620 pH/Blood Gas Analyzer, Instrumentation Laboratory, Lexington, MA, USA). Lactate (Lac), sodium (Na), potassium (K), chloride (Cl) and ionized calcium (iCa) ions were measured (ABL555, Radiometer Danmark) and apparent Strong Ion Difference (SIDa) was calculated as
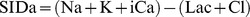
Glucose and hemoglobin concentration were also measured (ABL555, Radiometer Danmark).

#### Hemodynamic and respiratory parameters

Hemodynamic parameters (arterial blood pressure and heart rate) and ventilator settings (respiratory rate, tidal volume, positive end-expiratory pressure, mean airway pressure) were recorded throughout the protocol. Oxygenation was studied by arterial blood gas analysis (1620 pH/Blood Gas Analyzer and 682 CO-Oxymeter, Instrumentation Laboratory, Lexington, MA, USA).

#### Lactate pharmacokinetics

Lactate wash-out kinetics was studied in the animals where lactate infusion was interrupted after randomization (transient groups: group T and group T+B).

Lactate kinetics was studied using a model previously described [26]. On the base of lactate increase over the first 75 minutes of infusion, clearance of exogenous lactate and basal lactate production were calculated. Lactate clearance (ml/kg/min) was calculated as the ratio between the lactate load (mmol/kg) and the area under the lactate concentration curve over time (mmol/min/l). We considered endogenous lactate production constant over the first 75 minutes of lactate infusion and we calculated basal lactate production (µmol/kg/min) as the product of basal lactate concentration (Lacbasal - time 0) and exogenous Lac clearance.

Lactate pharmacokinetics was assessed using the model:
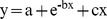
fitting, animal per animal, lactate concentrations (y) and time (x), where time 0 was considered time 75. The fitting was performed by means of the least squares method using Sigma Stat software (Systat Software, Inc.). From the fitting analysis coefficient b and c were derived animal by animal; using coefficient b, half time decay (T½) was calculated as 1/b. Coefficients and time decay were then compared for statistical significance.

#### Phosphofructokinase (PFK) activity

After the analysis of the first 32 randomized animals, we conducted a new set of experiments to better interpret our results. Eleven animals were randomized to receive the lactic acid load as previously described. Three animals were sacrificed soon after the acid load; 4 animals received transient infusion of lactic acid+sodium bicarbonate infusion (group T+B) and 4 the acid load without sodium bicarbonate infusion (group T). Oxidative soleus muscle (MS) and glycolytic extensor digitorum longus (ME) were then collected and stored with snap freezing technique for PFK activity analysis. Enzymes' activities were also determined [27]. Protein concentration was measured according to Lowry [28], and the PFK enzymatic activity was expressed as µmol/min/mg of protein.

### Statistical analysis

Results are expressed as mean ± SEM. Analysis of variance was conducted and Bonferroni test was used for all pair-wise comparisons, when indicated. To compare two groups of variables T-test was used or Mann-Whitney Rank Sum Test if normality test failed. Least square linear regression analysis was used to correlate variables. Multiple linear regression was used to correlate base excess and lactate, animal by animal. Statistical significance was accepted as P<0.05. Analysis was performed with the SAS System for Windows version 9.1, unless otherwise specified.

## Results

### Effects of lactic acid load

#### Acid-base parameters

**Figure 2:**
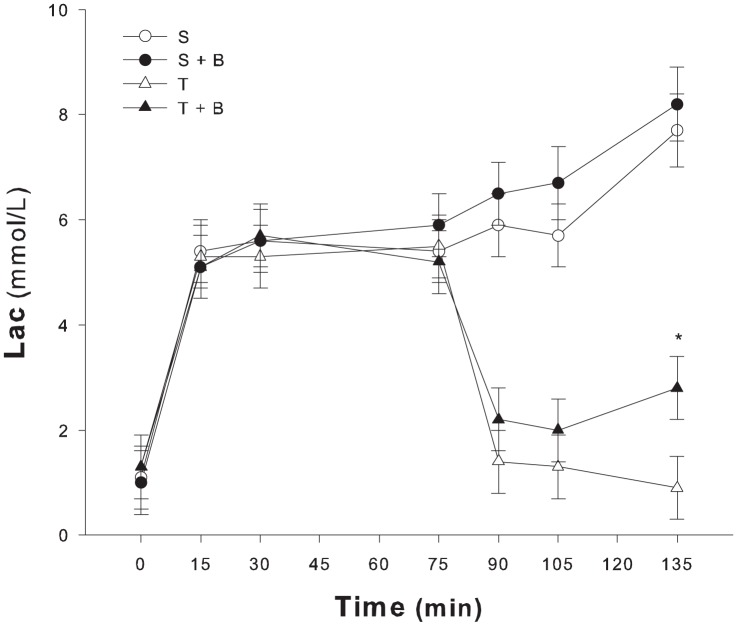
Lactate over time in the four groups. Lactic acid load caused blood lactate to rise in all groups (^#^ P<0.05 vs. time 0). After 135 minutes in the sustained groups blood lactate remained high both in the sustained (S) and the sustained+NaHCO_3_ (S+B) group. In the transient (T) groups blood lactate levels after 135 minutes were different from values at time 75 minutes (° P<0.05). Animals that received NaHCO_3_ (T+B) had higher lactate levels (* P<0.05 vs. transient group).

**Figure 3:**
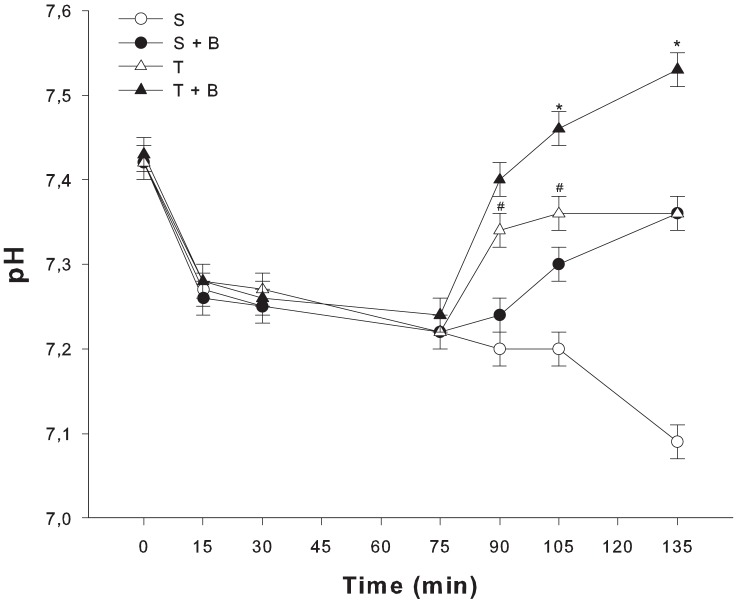
Blood pH over time in the four groups. After 75 minutes of infusion of lactic acid blood pH drop in all groups (^#^ P<0.05 vs. time 0). At 135 minutes pH normalized in the transient group (T) while overshoot to alkalemic values when animals received NaHCO_3_ (T+B). In the sustained group (S) pH continued to drop while alkaline infusion (S+B) resulted in correction of acidosis. ° P<0.05 vs time 75; * P<0.05 vs. control.

The infusion of lactic acid caused lactate to rise (1.2±0.07 mmol/l to 5.5±0.23, P<0.05 time 0 vs. 75, Figure 2) and pH to drop (7.426±0.005 vs. 7.227±0.009, P<0.05, Figure 3). SIDa decreased (31.92±0.54 mEq/l vs. 25.27±0.88, P<0.05) and hemoglobin was significantly lower (13.5±0.3 mg/dl vs. 10.9±0.3, P<0.05). Hyperchloremia (107±0.6 mEq/l vs. 112±0.7, P<0.05) and hypercapnia (40.2±0.6 mmHg vs. 51.2±1.5, P<0.05) also developed. Base excess decreased from 2.08±0.5 mmol/l to −6.3±0.8 (P<0.05). Changes of base excess correlated with changes of lactate (R2 = 0.81, P<0.05, multiple linear regression). Data are presented in Table 1.

**Table 1:** Acid base variables and plasma chemistry.

	Min	S	*S+B*	T	*T+B*
**pH**	0	7.42±0.01	7.42±0.01	7.43±0.01	7.43±0.01
	75	7.22±0.02^a^	7.22±0.02^a^	7.23±0.02^a^	7.24±0.01^a^
	135	7.13±0.05^a^	7.36±0.04^b^ ^c^	7.36±0.02^b^	7.53±0.03^a^ ^b^ ^c^
**pCO_2_** (mmHg)	0	40±1.1	39±0.9	42±1.3	40±1.1
	75	55±3^a^	49±2.4	55±2.9	47±3.4
	135	56±4.9^a^	55±4.6^a^	46±2.8	49±4.9
**HCO_3_** (mmol/l)	0	26.2±0.9	25.3±1	27.5±1	26.9±0.8
	75	22.7±1.6	19.9±1	22.8±1.4	20.0±1.6
	135	19.3±2.6	31.8±4.1^b^ ^c^	25.9±1.5	40.5±3^a^ ^b^ ^c^
**BE** (mmol/l)	0	1.7±1.1	0.8±1.1	3.2±1.1	2.6±0.9
	75	−5±1.9	−7.9±1.1	−4.8±1.6^a^	−7.3±1.7^a^
	135	−10.2±3.5^a^	6.3±4.7^b^ ^c^	0.5±1.6	17.4±2.7^a^ ^b^ ^c^
**Lac** (mmol/l)	0	1.2±0.2	1.1±0.1	1.3±0.1	1.3±0.1
	75	5.4±0.5^a^	6±0.5^a^	5.5±0.4^a^	5±0.5^a^
	135	7.4±1.6^a^	8±1.5^a^	1±0.2^b^	2.9±1^b^ ^c^
**Na** (mEq/l)	0	136.2±3.8	138.8±2.5	137.9±1.3	135±1.5
	75	132.4±2.8	136.2±3.3	138.2±1.5	137.5±1.1
	135	135.3±1.9	144.3±1.1	136.4±1.7	147.8±1.2^a^ ^b^ ^c^
**K** (mEq/l)	0	4.26±0.16	3.64±0.09	3.94±0.17	3.86±0.17
	75	4.36±0.16	3.75±0.2	4.18±0.25	3.9±0.25
	135	4.73±0.31	3.95±0.45	4.8±0.42	4.36±0.31
**Cl** (mEq/l)	0	105.3±2.4	109.0±0.7	105.8±0.7	108.0±0.9
	75	110.7±2.3	112.5±1.2	113.2±1.6^a^	112.3±1
	135	110.6±3	112.0±1.5	112.6±0.8^a^	107.3±1.8^c^
**SIDa** (mEq/l)	0	31.5±1.1	31.5±0.8	33.4±1.1	30.8±1.1
	75	27.1±2.2	23.2±1.4^a^	25.5±2.6^a^	25.7±0.5
	135	29.8±2	30.9±3	32.2±0.8^b^	43.4±1.8^a^ ^b^ ^c^
**iCa** (mEq/l)	0	0.94±0.13	0.99±0.1	0.98±0.07	0.99±0.07
	75	0.93±0.13	1.07±0.09	1.06±0.06	1.08±0.06
	135	1.02±0.08	0.97±0.11	0.96±0.07	0.85±0.07
**Glc** (mg/dl)	0	165±18	153±13	149±8	160±17
	75	125±6	116±10	120±7	112±8^a^
	135	116±15	117±12	112±6^a^	79±13^a^
**Hb** (g/dl)	0	13.4±0.6	14±0.8	13.4±0.5	13.3±0.4
	75	10.9±0.7	11.1±0.8	10.8±0.7^a^	10.8±0.6
	135	7.7±0.5^a^	8.7±1.4^a^	9.9±0.4^a^	9.1±0.5^a^

ANOVA P<0.05:

a vs. 0,

b vs. 75,

c S vs. S+B or T vs. T+B.

BE, base excess; Lac, lactate concentration; Na, sodium concentration; K, potassium concentration; Cl, chloride concentration; SIDa, stron ion difference; iCa, ionized calcium concentration; Glc, glycemia; Hb, hemoglobin concentration.

Data are expressed as mean ± SEM.

#### Hemodynamic and respiratory parameters

During the primed infusion of lactic acid 11 animals developed severe hemodynamic instability and were excluded. Three animals died soon after randomization (one in group S, two in group S+B) and were replaced in the randomization process so that a total of 32 normoxic and normotensive rats completed the randomization process (n = 8 per group). At baseline, excluded and randomized animals were similar in terms of weight, surgical time, respiratory, acid base and hemodynamic variables except for a trend towards higher values of lactate (1.23±0.07 mmol/l vs. 1.49±0.11, P = 0.056 randomized animals vs. animals who failed lactic acid infusion, respectively), and a significantly higher heart rate (482±17 vs. 431±8 bpm, P<0.05).

### Effects of randomization

#### Acid-base parameters

After the randomization, pH drop over time during the sustained infusion of lactic acid. There was a non significant increase of lactate levels. In the transient groups (T and T+B) 15 minutes after the end of lactic acid infusion, blood lactate concentration, pH and BE values were normal.

When NaHCO_3_ was infused, pH normalized in the group with sustained infusion (group S+B: from 7.22±0.02 to 7.36±0.04, P<0.05) while rose to alkalemic values in the transient group (group T+B: from 7.24±0.01 to 7.53±0.03, P<0.05). Effects on pH were mainly related to sodium dependent changes of SIDa: Na^+^ increased from 136.2±3.3 mEq/l to 144.3±1.1 and from 137.5±1.1 to 147.8±1.2 in the sustained group (S+B, P<0.05) and in the transient group (T, P<0.05), respectively.

#### Hemodynamic and respiratory parameters

As shown in Table 2, through the experimental time, mean arterial pressure and heart rate were similar in sustained and transient lactic acidosis groups. Sodium bicarbonate infusion did not modify hemodynamic parameters.

**Table 2:** Hemodynamic and respiratory variables.

	min	S	*S+B*	T	*T+B*
**Mean arterial pressure** (mmHg)	0	134±6.8	132±3.92	134±7.51	133±4.76
	75	140±5.6	131±5.59	134±6.96	139±5.85
	135	108±11.96	103±17.13	119±7.29	113±10.73
**Heart rate** (beats/min)	0	417±20.77	460±13.55	439±12.21	417±15.88
	75	382±10.84	390±19.82	389±5.9	368±15.18
	135	351±22.03	404±8.7	394±18.73	397±21.99
**Tidal Volume** (ml)	0	2.81±0.14	3.03±0.19	2.78±0.12	3.03±0.08
	75	2.81±0.14	3.03±0.19	2.78±0.12	3.03±0.08
	135	2.81±0.16	3.03±0.25	2.78±0.12	3.03±0.08
**Respiratory rate** (breaths/min)	0	72±2.1	64±3.8	70±1.6	69±2.7
	75	71±2.2	64±3.7	71±1.5	69±2.6
	135	71±2.5	64±5.4	70±1.6	70±2.5
**Mean airway pressure** (cmH_2_O)	0	6.4±0.2	7.4±0.51	6.5±0.37	6.7±0.4
	75	6.5±0.24	6.9±0.55	6.5±0.27	7.6±0.8
	135	7.1±0.42	6.7±0.27	6.9±0.25	6.8±0.2
**Oxygenation** (PaO_2_ - mmHg)	0	300±14.32	314±13.92	328±9.19	295±14.04
	75	277±17.84	281±18.44	298±12.29	273±11.22
	135	269±31.4	216±36.12	332±15.87	272±11.69^c^

ANOVA P<0.05:

c S vs. S+B or T vs. T+B.

Data are expressed as mean ± SEM.

Ventilator parameters were unchanged and were similar among randomization groups. At the end of the 135 minutes, animals that received sodium bicarbonate infusion were more hypoxemic then their relative controls: 216±36.12 mmHg vs. 269±31.4, group S+B vs group S (P = 0.0511) and 272±16.96 mmHg vs. 332±15.87, group T+B vs group T (P<0.05).

### Metabolic measurements

#### Lactate pharmacokinetics

As shown in Figure 2, at the end of the protocol lactate was significantly higher in the T+B group than in the T group.(2.9±1 mmol/l vs. 1±0.2, group T+B vs. group T respectively, P<0.05 – Table 1). Despite similar endogenous lactate production and clearance, the decay of lactate over time was different when bicarbonate was added (Table 3).

**Table 3:** Lactate pharmacokinetics.

	T	*T+B*
**Lac_basal_** (mmol/l)	1.3±0.1	1.3±0.1
**Lac clearance** (ml/kg/min)	13.3±0.8	14.5±1.1
**BLP** (µmol/kg/min)	17.65±1.2	18.4±1.4
**Lac_75′_** (mmol/l)	5.5±0.4	5.2±0.5
**b**	0.354±0.106	0.19±0.101^a^
**T ½** (min)	4.294±0.736	17.983±7.592^a^
**c**	−0.0589±0.038	0.116±0.058^a^

Mann-Whitney Rank Sum Test P<0.05:

a T+B vs. T.

Lac_basal_, lactate concentration at baseline; Lac clearance, clearance of exogenous lactate; BLP, basal lactate production; Lac_75′_, lactate concentration at time 75; b, coefficient b; T ½, half time decay; c, coefficient c.

Data are expressed as mean ± SEM.

**Figure 4:**
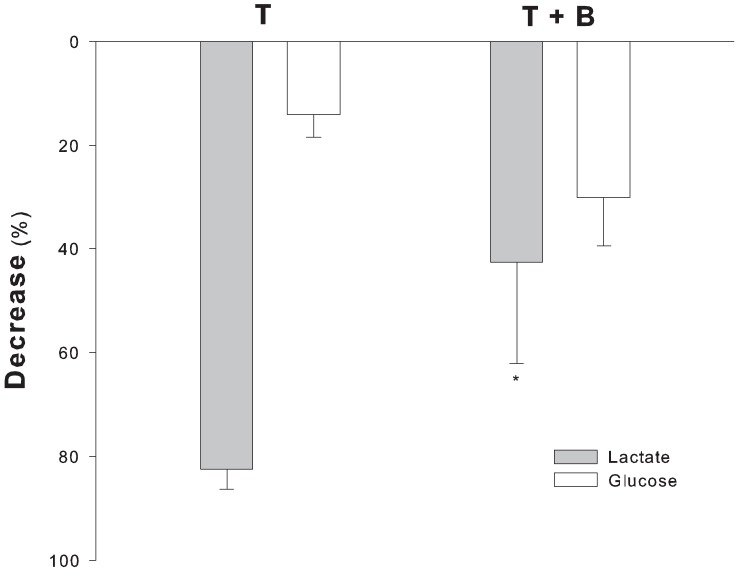
Blood lactate and blood glucose decrease in transient lactic acid infusion group. Decrease over time of blood lactate concentration and glycaemia in the animals with transient lactic acid infusion. Values are expressed as percentage decrease from time 75 to 135 (*P<0.05 vs. transient group).

When sodium bicarbonate was infused (group T+B) blood glucose concentration slightly decreases over time (112±8 mg/dl vs. 79±13, time 75 vs. 135 respectively, P = 0.095 – Table 1). Glucose and lactate decay was different in T and T+B groups (Figure 4). Glucose changes over time inversely correlated with changes of lactate (R^2^ = 0.582, P<0.05): the higher the changes of lactate, the lower the glucose changes.

#### PFK activity

**Figure 5:**
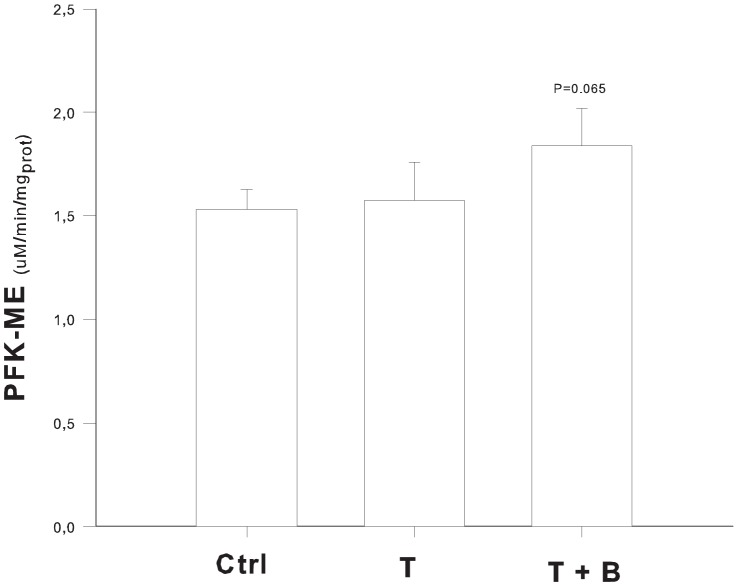
PFK activity. Activity of the glycolytic enzyme phosphofructokinase (PFK) in the glycolitic muscle (ME- extensor digitorum longus) was slightly higher when bicarbonate was infused (P = 0.067). Ctrl = end of lactic acid infusion (i.e 75′ after the start of acid load).

The activity of PFK in the oxidative soleus muscle was similar in the studied groups (1.675±0.171 µmol/min/mg of proteins, P = 0.11). Conversely, PFK activity in the glycolytic extensor digitorum longus muscle was higher when bicarbonate was infused (P = 0.067, Figure 5). The higher the blood pH measured before muscle harvest the higher the activity of PFK, as shown by linear regression analysis (R^2^ = 0.475, P<0.05).

## Discussion

We set up an experimental model of transient versus sustained lactic acid infusion in normoxic and normotensive rats to investigate the systemic effects of lactic acidemia per se without the confounding factor of an underlying organic acidosis. The load of lactic acid caused acidemia of both metabolic and respiratory origin. These effects quickly reversed during the transient infusion. Bicarbonate treatment allowed to normalize acid-base parameters during the sustained infusion of lactic acid, but led to overshoot alkalization during the transient load of acid that affected lactate washout kinetics and glucose metabolism.

A number of animal experiments have been conducted to investigate lactic acidosis. As opposed to other more clinically relevant models such as hypoxia [16]–[18]; [29], sepsis [30]; [31], hemorrhage [20], phenformin intoxication [15]; [32] or hepatectomy [32], in this study we used an infusion of lactic acid in normoxic and normotensive animals to titrate systemic blood lactate concentrations and pH. While alkali treatment during hypoxia or hemodynamic instability may interfere with the cause and the systemic effects of the underlying acidemia, our model allowed to investigate changes of pH and lactate kinetics without possible confounding variables such as oxygen delivery impairment, mitochondrial defects or abnormalities of lactate clearance.

The effects of lactic acid load in our experiments were straightforward: pH consistently drop down to less than 7.2, while lactate rose to clinically significant levels. The acidemia that developed was of mixed origin: despite hemodiluition and hyperchloremia, pH changes were mainly due to lactate, as shown by the correlation between base excess drop and lactate rise. However a respiratory contribution to the drop of pH was also evident, whereas minute ventilation was unchanged throughout the protocol. In line with previous experiences [15], the induction of lactic acidemia resulted in a degree of mortality rate. However, at the beginning of randomization animals included in the randomization process were normoxic and normotensive.

After the randomization, pH drop over time during the sustained infusion of lactic acid. Even if not significant, there was a rise in lactate levels. Since PaCO_2_ and electrolyte concentrations did not change through the randomization, lactate increase may be interpreted as an extra load of organic acid.

Sodium bicarbonate treatment caused pH to rise. Alkalinization mainly occurred because of a sodium dependent change of SIDa, with some possible contribution of the reduction of weak acids due to hemodilution. The role of respiratory alkalosis was negligible, consistent with the fact that the release of CO_2_ is known to occur early after the infusion of sodium bicarbonate and depends on both the infusion rate and the concentration of non-bicarbonate buffers [33]–[35] that was relatively low during the randomization time.

Although the starting concentration of bicarbonate was similar in S+B and T+B groups, pH normalized during sustained infusions of lactic acid (from 7.22±0.02 to 7.36±0.04) while increased up to alkalemic values (from 7.24±0.01 to 7.53±0.03) when acid latic infusion was transient. Re-perfusion of hypoperfused or ischemic territories is characterized by an organic acid washout and a transient acid load, that cause a transient pH decrease. In this case sodium bicarbonate infusion may exceed the desired effect of reversing acidemia. On the contrary, the normalization of pH during sustained lactic acid infusion seems to be relevant, even if we did not find hemodynamic instability at low pH that many physicians advocate to start alkalinization therapy [25].

As expected, sodium significantly increased in both groups treated with bicarbonate. We also observed in these groups an oxygenation decrease probably due to a fluid load.

Bicarbonate infusion affected blood lactate levels differently during sustained or transient acidemia. In fact, lactate slightly rose in the S+B and S groups, but in a similar fashion. On the contrary, when the acid load was transient, at the end of the experiment lactate was significantly higher in the group of animals that received bicarbonate treatment (T+B). The abrupt and wide change of pH that followed bicarbonate infusion in this group possibly affected lactate metabolism, given the modulatory role of pH on blood levels of lactate [15]–[18]; [20]; [31]. Pharmacokinetic results suggest a reduction of the oxidation of lactate after bicarbonate infusion, according to Chiolerò et al. [26]. Lactate kinetics are also in line with those from Druml et al. who found that respiratory alkalosis decreases the clearance of infused lactic acid [36], and Abu Romeh et al. who found in a rat model of hypoxic lactic acidemia that systemic acidosis inhibits net lactic acid production. [29] Because it is known that pH modulates both glycolitic flow [37]–[40] and lactate cellular uptake [41]–[44], and because lactate undergoes preferential oxydation when in excess [45], we speculate that lactate was preferentially oxidated at low pH. On the contrary, when bicarbonate was infused, alkalosis favored glucose metabolism so that glucose levels decreased more than lactate and lactate half-life increased. The data on PFK activity seem to confirm this hypothesis.

Provided the effects of bicarbonate infusion on pH differed under a persistent or transient acid load and alkalization affected the rate of lactate disposal during the transient acid load, when deciding to infuse sodium bicarbonate one should take into consideration the metabolic effects of pH on the cell and the possible consequences on adaptation to energy failure [44]; [46]–[50].
